# Effectiveness of a community paramedic-led health assessment and education initiative in a seniors’ residence building: the Community Health Assessment Program through Emergency Medical Services (CHAP-EMS)

**DOI:** 10.1186/s12873-017-0119-4

**Published:** 2017-03-09

**Authors:** G. Agarwal, R. Angeles, M. Pirrie, F. Marzanek, B. McLeod, J. Parascandalo, L. Dolovich

**Affiliations:** 1 Departments of Family Medicine, Clinical Epidemiology and Biostatistics, Quality Assurance Program Coordinator for Family Medicine Residency, Residency Program Research Coordinator, Family Medicine Residency Program, McMaster University, 100 Main Street West, 5th Floor, Hamilton, ON L8P 1H6 Canada; 2 Hamilton Paramedic Services, City of Hamilton, Canada

**Keywords:** Community paramedicine, Intervention, Elderly, Diabetes risk, Cardiovascular disease, Falls, Low income

## Abstract

**Background:**

Seniors living in subsidized housing have lower income, poorer health, and increased risk for cardiometabolic diseases and falls. Seniors also account for more than one third of calls to Emergency Medical Services (EMS). This study examines the effectiveness of the Community Health Assessment Program through EMS (CHAP-EMS) in reducing blood pressure, diabetes risk, and EMS calls.

**Methods:**

Paramedics on modified duty (e.g. injured) conducted weekly, one-on-one drop-in sessions in a common area of one subsidized senior’s apartment building in Hamilton, Ontario. Paramedics assessed cardiovascular, diabetes, and fall risk, provided health education, referred participants to local resources, and encouraged participants to return to CHAP-EMS for follow-up. Reports were faxed to the family physician regularly. Blood pressure was collected throughout the one year intervention, while diabetes risk was assessed at baseline and after 6–12 months. EMS call volumes were collected from the Hamilton Paramedic Service for two years pre-intervention and one year during the intervention.

**Results:**

There were 79 participants (mean age = 72.2 years) and 1,365 participant visits to CHAP-EMS. The majority were female (68%), high school educated or less (53%), had a family doctor (90%), history of hypertension (58%), high waist circumference (64%), high body mass index (61%), and high stress (53%). Many had low physical activity (42%), high fat intake (33%), low fruit/vegetable intake (30%), and were current smokers (29%). At baseline, 42% of participants had elevated blood pressure. Systolic blood pressure decreased significantly by the participant’s 3^rd^ visit to CHAP-EMS and diastolic by the 5^th^ visit (*p* < .05). At baseline, 19% of participants had diabetes; 67% of those undiagnosed had a moderate or high risk based on the Canadian Diabetes Risk (CANRISK) assessment. 15% of participants dropped one CANRISK category (e.g. high to moderate) during the intervention. EMS call volume decreased 25% during the intervention compared to the previous two years.

**Conclusions:**

CHAP-EMS was associated with a reduction in emergency calls and participant blood pressure and a tendency towards lowered diabetes risk after one year of implementation within a low income subsidized housing building with a history of high EMS calls.

**Trial registration:**

Retrospectively registered on May 12^th^ 2016 with clinicaltrials.gov: NCT02772263

## Background

Seniors are frequent users of health care resources [1], particularly seniors with low socio-economic status and poor health [2]. Older adults with high comorbidity (>3 chronic conditions) report worse health, take more prescription medications and have the highest rate of health care visits [1]. Older adults account for more than a third of all Emergency Medical Services (EMS) calls [3–6]. The majority of EMS calls are related to cardiopulmonary conditions, diabetes, and falls related trauma [3–6]. Older adults living in subsidized housing units report poorer health from a multitude of chronic illnesses, such as cardiovascular disease (CVD) and diabetes, compared to those living in unsubsidized housing units [7–9]. Thus, it is prudent to target this population with health promotion and disease prevention initiatives.

CHAP-EMS is a low-cost, community paramedicine program designed to assess community dwelling seniors for lifestyle risk factors that may impact their health and wellbeing and to provide targeted education to address the pertinent risk factors [10]. Each participant is guided through a structured health-risk assessment focused on diabetes, CVD and other potential health issues. The assessment is conducted by a trained paramedic in a common area within the community housing building. Data gathered are used to develop individualized action plans concerning health-risk reduction, to direct participants to local health activities or resources, educate on promoting health and to transmit this information to a participants’ family physician. There a number of key factors that make CHAP-EMS appealing as a health service delivery program. The program utilizes paramedics on modified duties; these are paramedics who are unable to work their regular duties due to health-related concerns. The program focuses on locations (residential buildings) that generate a high volume of EMS calls. CHAP-EMS creates strong program-community partnerships so as to leverage already available health care services and create better links to those services. Finally, the program generates clinically useful health data and communicates that data to those who can best use it to improve a person’s health - the person themselves and their family physician.

The goal of CHAP-EMS is to prevent emergency events before they occur by increasing older adults’ awareness of their own health risk factors, encouraging them to manage these risk factors, and linking them with locally available community resources and services based on those risk factors. Furthermore, since the participants’ health information (with their consent) is sent to their primary care physician, health care services (promotion, screening, and follow-up) can be further coordinated to improve the participant’s health status.

The objective of this paper was to evaluate whether a weekly 8-hour CHAP-EMS program was associated with changes in (1) number of emergency EMS calls (9-1-1) from the seniors’ residence building, (2) mean blood pressure (BP) of participants and (3) diabetes risk profile of participants after one year of implementation.

## Methods

### Design

A prospective pre-post approach was used for this intervention study. The CHAP-EMS program was implemented from January 1, 2013 to December 31, 2013. The program was piloted between November and December 2012 to ensure fidelity of the program before the full evaluation was conducted.

### Setting and participants

The study took place in a seniors’ housing building in Hamilton (a city in Ontario, Canada, population 520,000), [11] which was comprised of 260 apartments units, 84.5% of which were occupied by seniors aged 65 or older. Most occupants were assessed as being of low income and, accordingly, were receiving rent subsidies from the City of Hamilton Housing department. The building was identified by Hamilton Paramedic Services as having an unusually high EMS call rate and was considered an ideal location for the placement of the community health assessment program. Eligible study participants were all building residents over 65 years old. The program was delivered in a community common room area at a small informal station.

### Intervention

The CHAP-EMS program was delivered by two trained paramedics for eight hours a week (four hours per session, both sessions were on the same day). A more detailed description of our intervention is published elsewhere. [10] The paramedics were trained by a public health nurse and family doctor to take blood pressure, conduct health assessments, educate residents, and utilize the CHAP-EMS database. Building residents were invited to participate in the CHAP-EMS program through posters/flyers and announcements in tenant meetings delivered by members of the research team and paramedics. After providing written consent, participants were guided through a 15–20 min defined risk assessment by a trained paramedic. Risk factors assessed were those related to cardiovascular and diabetes risk (BP, diabetes-risk status, lifestyle factors), and potential for falls. Blood pressure was taken using the automated WatchBP Office (which takes 3 measurements on the same arm, on seated participants, and averages the results) following the Hypertension Canada recommended procedure [12]. All information was entered in the CHAP-EMS database which summarized the risk factor information based on pre-specified algorithms. Based on these, the paramedic provided education and developed an individualized action plan directing participants to use available community resources to assist them in addressing their risk factors. All participants received a copy of their risk profile, relevant health education, and information on local resources. They were advised to return to CHAP-EMS sessions regularly for BP monitoring and follow-up, and to further address risk factors in an ongoing fashion. Each participant’s information was faxed to his/her family physician once a month. Participants were also given cards containing a record of all BP readings to use for self-monitoring and sharing with their family physicians. Participants who had moderate or high risk of developing diabetes were advised to return for a fasting capillary blood glucose (CBG) test at the next visit. Participants were advised to see their physician if they had high CBG test results. A standard protocol was followed for referral or emergency action when participants had alarming findings (very high or low BP or CBG).

### Data collection

BP was collected using the Watch-BP Office (a Canadian Hypertension Society validated machine). Diabetes risk was generated using the Canadian Diabetes Risk (CANRISK) questionnaire [13], which asks 12 questions and will determine a risk category of either low, moderate or high for developing diabetes in the next 10 years. Cardiovascular risk factors were collected by asking about lifestyle details, such as dietary habits (including salt intake, fast food and fat intake). These questions were obtained from the established CHAP Program of research [14], and standard Canadian Community Health Survey. [15] Risk of falls was assessed using the Timed Up and Go (TUG) test [16]. Demographic information, BP values, CANRISK scores, and data about modifiable risk factors as recorded in the CHAP-EMS database were collated for each individual, from each session. EMS emergency (911) call rates specific to the intervention building were obtained from the routinely collected statistics held by the Hamilton Paramedic Service for the study year (2013) and the two preceding years (2011, 2012) to allow trends to be examined. This information was collected by the 911 call dispatch centre by postal code, as well as separately on forms that each ambulance team completed after a call. These two sources of data were aggregated and reconciled.

### Power calculation

The power was computed post-hoc since the study was initially conducted to determine the feasibility of building resident participation in the program. The intention was to detect a change of 5 mmHg in both the systolic and diastolic BP (SD = 10 mmHg) because this has clinically significant implications [17]. With the 79 residents who participated in the study, there was a power of 0.88 to detect the desired change in BP.

### Analysis

All seniors who were 65 years and older and visited the CHAP-EMS program at least once were included in the analysis for the BP and CANRISK changes. Although the target of the CHAP-EMS program was older adults, any building resident who attended the program was assessed and received feedback, but data for those younger than 65 years were not analyzed. The change in the EMS emergency (911) call rates was based on all calls coming from the subsidized building whether or not the callers participated in the CHAP-EMS study, so providing the program to younger residents could have had an effect on this outcome. Data regarding call numbers was collected prospectively during the implementation period, and had been retrospectively collected for the two year period prior to implementation; duplicate calls were removed and individual visits only were accounted for in the statistical analysis.

Demographic characteristics and modifiable risk factors of the attendees were analyzed using descriptive statistics. Hierarchical linear modelling of BP was conducted to assess for the changes in systolic and diastolic BP over time within the same individual. We also analyzed the number of visits for normalisation of BP according to the 2015 Canadian Hypertension Education Program Guidelines, aiming for the goal of 140/90 [18]. Baseline descriptive analysis of the CANRISK scores was performed, as well as a comparison with the descriptive analysis of CANRISK scores obtained 6 months after the initial visit. Preliminary post-hoc cost analysis, including a sensitivity analysis, was performed using referenced costs from the literature for ambulance transport [19, 20], EMS time and hospital visits [21–23], associating these with reductions in EMS calls, to assist in the interpretation of the results.

## Results

During the one year program implementation, 79 of 234 (34.8%) eligible residents participated in the CHAP-EMS program. Forty-eight (61%) participants had two or more visits to the program (Median = 15.5, Range 1–59). There were a total of 1,365 participant visits to the sessions with an average of 20 participants (SD = 9) per session. Generally, there were three to five new participants enrolled in the program every month. Thirty-four family physicians were contacted and asked if they wanted to participate with the CHAP-EMS program and receive information collected through its database, with consent of the respective participant; 26 agreed to participate by receiving the information via fax. Others did not reply to our request.

### Characteristics of participants

Table 1 summarizes the participant characteristics and results of the risk factor assessments. The mean age of the participants was 72.2 years. Most participants were female (68.1%) and 90.3% had a family doctor. The number of medications taken by the participants daily ranged from 0 to 12 medications (Mean 6.7, SD 4.2).

**Table 1:** Characteristics of participants attending CHAP-EMS sessions

Participant Profile	N = 79
Mean Age, in years (SD)	72.2 (12.1)
% Female	68.1
Education (%)	
• Some high school or less	33.3
• High school Diploma	19.4
• Some college of more	29.2
• Not specified	17.1
Lives alone	96.2
% with Family Doctors	90.3
% Self-reported as previously diagnosed with Hypertension	58.3
% Self-reported as previously diagnosed with Diabetes	19.0

### EMS calls from the seniors’ residence building

In 2011, prior to any program implementation, 114 emergency (9-1-1) calls were received from the study site. In 2012, a modified version of the program looking only at BP had run for a few months in the latter portion of the year, as the program was developed and refined; the number of emergency calls received was 104. In 2013, the CHAP-EMS program was fully implemented for the duration of the entire year and the number of emergency calls dropped to 84. Compared to the average number of emergency calls in the building in the previous 2 years (2011 and 2012), the number of emergency calls decreased by 7.1% (CI: 3.1–12.5%) at the 6 month mark, and 25% (CI: 17.3–34.0%) after a full year of CHAP-EMS programming (2013).

### Modifiable risk factors

At baseline, the most prevalent risk factors displayed by the participants were high waist circumference (63.9%) and elevated body mass index (61.1%). These risk factors are associated with the other prevalent risk factors, such as low physical activity (41.7%), high fat food intake (33.3%) and low fruit and vegetable intake (29.6%). Over half of the participants (52.8%) also indicated that they felt stressed most days. Nearly one third of participants were smokers (29.2%).

### Blood pressure

Table 2 shows the mean systolic and diastolic BP of participants attending the CHAP-EMS during the first five visits. The results show that there was a trend of decreasing BP over time. There was a significant drop in the systolic BP and diastolic by the 3^rd^ and the 5^th^ CHAP-EMS visit respectively.

**Table 2:** Blood pressure (BP) of participants during the first 5 CHAP-EMS sessions

Visit	Systolic BP (SE)	Diastolic BP (SE)
1	136.9 (2.6)	78.1 (1.2)
2	135.4 (2.7)	77.4 (1.3)
3	132.2 (2.5)*	75.5 (1.4)
4	131.4 (2.8)*	75.4 (2.0)
5	120.2 (2.8)*	74.4 (1.6)*

*Significant decrease compared to Baseline *p* < 0.05

**Fig. 1:**
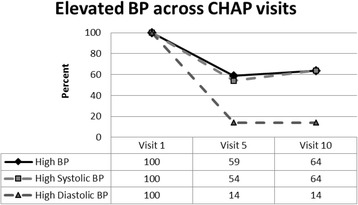
Normalization of BP among participants who had elevated BP during visit 1

Overall, 41.7% of the participants had elevated BP (either systolic or diastolic, >140/90 respectively) during the initial visit. Of participants previously diagnosed with hypertension, 53.5% had elevated BP. Of participants not previously diagnosed with hypertension, 40.7% had elevated BP. Of those who had elevated BP at the initial visit (n = 27), 41% had BP that was within the normal range by visit 5, and 36% by visit 10 (see Fig. 1). Of those presenting with elevated systolic BP at visit 1 (n = 26), 46% had normalized systolic BP by visit 5 and 36% by visit 10; of those presenting with elevated diastolic BP at visit 1 (*n* = 8), 86% had normalized diastolic BP at both visits 5 and 10.

### CANRISK score

Among the 79 participants, 15 (19.0%) already had diabetes while 66.7% had moderate or high risk of developing it based on the CANRISK assessment. Of those who repeated the CANRISK (n = 26) after a minimum of 6 months and a maximum of the 1 year intervention period, 15% dropped from the high risk category to moderate risk or from moderate risk to low risk (Wilcoxon P test, *p* = 0.180). Though this was not statistically significant it was a reduction in the intended direction.

Many people who had completed the CANRISK assessment and were at risk of developing diabetes or already had it, also had elevated BP at the initial visit (either systolic or diastolic > 130/80 respectively; see Table 3). Though not statistically significant there was a tendency towards normalization of BP across the ten visits, in people who had a moderate/high CANRISK score.

**Table 3:** Blood pressure (BP) status in those at risk of diabetes or with diabetes already, by visit number

Diabetes status	N	% Elevated BP (95% CI)		
		Visit 1	Visit 5	Visit 10
Diabetes prior to study	15	66.7 (33.3–100.0)	57.1 (16.7–97.6)	66.7 (0.1–100.0)
Moderate-High CANRISK	42	38.8 (25.0–52.6)	34.8 (20.4–49.3)	27.3 (11.8–42.7)

### Follow-up during the CHAP EMS sessions

Participants who had specific risk factors were referred to available resources (exercise programs, food and nutrition resources, Community Care Access Centre (CCAC), social welfare organizations, public health, etc.). Most of the regular participants were referred to and enrolled in the in-house wellness program run by the Municipal authorities, which included an exercise program and scheduled lectures. Participants who were noted to have elevated BP were referred to see their family physicians. Three participants who were previously undiagnosed returned with a diagnosis of hypertension and were subsequently treated. Five participants previously diagnosed with hypertension had their medications adjusted. Furthermore, because of risks identified from the CANRISK assessment, one participant was confirmed to have diabetes by their family physician and two participants had their diabetes medications adjusted. Participants confirmed by the TUG test to be at risk of falls were referred to the CCAC for appropriate assistance, though we were unable to ascertain factual information about what happened subsequently due to confidentiality.

### Post-hoc cost analysis

Examining the literature, differing costs could be assumed following a 911 call. There could be an ambulance sent [costing between $240 (19) and $785 (20)] and there is often an emergency department (ED) transport [costing between $259(21) and $627 (22)] with an assessment in the ED [costing $842 (23)]. As per the literature, we assumed that once 911 is called in Ontario, an ED visit is mandatory [19]. Using the above references each paramedic visit and subsequent mandatory hospital ED physician evaluation were estimated to be an average of $1626, ranging from $499 as the lowest estimation of cost and $2254 as highest estimation of cost [19–23]. A sensitivity analysis demonstrated that the decrease of 20 EMS calls demonstrated by this pilot in one building, could decrease healthcare costs by as little as $9980 [19, 20] or as much as $45,080 per year [19, 20, 22], or by an average of $32,520. Paramedics staffing the CHAP-EMS program were on modified duties (e.g. due to injury), so they were being paid by the Paramedic Services but could not perform a traditional paramedic role due to health-related limitations. Therefore, there was no cost associated with having these paramedics on modified duty staff the CHAP-EMS program. The training for paramedics was provided at no cost for the pilot study. However, the estimated cost for training is nominal since it was a single 3-h training session, which has since been translated into an online training site, which is also free.

### Anecdotal evidence

Anecdotal narratives emerged from the program that add depth to our understanding of how CHAP-EMS assisted in the prevention of major morbidity for these participants. Some of these stories are related here.

A female participant previously thought to have had acid reflux, but with a history of angina, attended a CHAP-EMS session with acute sternal chest pain, which was diagnosed by the paramedics as severe angina requiring treatment and follow up. She was put in contact with a cardiologist who saw her that day and she was scheduled for balloon angioplasty within 2 weeks where upon her angina disappeared.

Another participant, an elderly male, had refused to call 911 but decided to seek help from the paramedics staffing CHAP-EMS since he was a regular participant. On attending and being assessed it was evident that he had acute chest pain, low BP and was feeling faint. An ambulance was called and he was transported to the ED whereupon he was diagnosed and treated for an acute myocardial infarction.

A female participant was well known to the paramedics and presented with dizziness and when her BP was taken was found to be bradycardic. She was transported by ambulance to the ED where a pacemaker was fitted by cardiology.

Another female participant known to be hypertensive had repeated high BP readings. Upon readings of 180/102 repeatedly one day, paramedics arranged for her to see her family physician that day, who increased her hydrochlorothiazide, added a new medication, and asked to continue being checked by CHAP-EMS.

## Discussion

The CHAP-EMS program was associated with a reduction in emergency calls and participant BP and a tendency towards lowered diabetes risk after 1 year of implementation within a low income subsidized housing building with a history of high EMS calls.

Emergency calls decreased by 25% during the year of CHAP-EMS, compared to previous years. This notable reduction in emergency calls has important health care resource implications, including human resource deployment and system costs. Though we were not able to conduct a formal economic analysis, our preliminary post-hoc cost analysis was performed to assist in the interpretation of the results. Taking into account local costs for EMS personnel and ambulance calls, a decrease of 20 EMS calls from just one building, as demonstrated possible by this pilot, was estimated to decrease healthcare costs by an average of $32,520 [19–23]. In addition to the CHAP-EMS goals (improving BP, decreasing lifestyle risk factors, decreasing emergency calls), anecdotal evidence demonstrated that the program was able to prevent serious morbidity by simply having the paramedic personnel on-site assessing emergency cases and coordinating emergency management. Therefore, the CHAP-EMS intervention holds promise in reducing the escalation of emergency calls and ED visits, and associated cost savings.

The World Health Organization has identified high BP as a leading risk factor for death [24]. A decrease of 10/5 mmHg reduces the risk of developing heart failure by about 50%, stroke by 38%, heart attack by 15%, and death by 10% [12]. On average, participants attending the CHAP-EMS sessions achieved a clinically meaningful reduction in BP by the 5^th^ visit. Furthermore, around 40% of the participants who had elevated BP during the first visit had a normal BP by the 5^th^ and 10^th^ visits (though less people attended for a 10^th^ visit than for a 5^th^). CHAP-EMS was associated with improvements in the BP of participants attending the sessions, most likely by diagnosing hypertension and initiating lifestyle changes and medication adjustments.

In Canada, 63% of adults with diagnosed hypertension also had diagnosed diabetes [25]. Literature states that 20% of diabetes cases remain undiagnosed [26] and diabetes is a risk factor for CVD and causes multiple organ system morbidity. To reduce the burden of CVD and diabetes, improved risk-factor screening has been recommended [27]. The CHAP-EMS program not only offered diabetes risk assessment but tailored lifestyle education. Though our sample was small, we noted a clinically important decrease in CANRISK risk category for 15% of the participating seniors that requires further study. Affecting any change in diet and exercise is a difficult goal and not easy to achieve. These individuals were followed for up to one year and their progress was good. It is possible that the constant presence of the CHAP-EMS program served as a reminder to achieve the lifestyle change they were trying to adopt. Alternatively, receiving tailored lifestyle advice that results in a greater knowledge of local programs and interventions may have precipitated change. Results showed that the attendees certainly had ample modifiable risk factors that could be targeted for change. As well as dietary and physical activity risk factors, smoking prevalence amongst this group was higher than the general population prevalence in Hamilton (29.2% versus 20%) [28].

Approximately 30% of the building’s residents visited the program over the year of implementation, which we consider to be a reasonable program penetration rate. Comparing to other community interventions, this participation level is very reasonable [29].

A systematic review in the United States of America comparing health promotion program participation rates showed that face-to-face participation rates can range from 3.1% for smoking campaigns, to 30% heart health and 55% general health promotion activities [29].

Paramedics have successfully held expanded roles that included both primary care health promotion and management, such as community education and engagement, preventive services, treatment of minor illness (for locally endemic conditions), and promotion of lifestyle change to prevent and manage chronic disease [30]. The paramedics working in the program were ‘modified’ and so were unable to assume traditional paramedic duties due to personal physical limitations, such as pregnancy or injuries. Despite these limitations, though rendering traditional paramedic duties unsuitable, the paramedic’s skill set is an incredible asset that can still allow for valuable health promotion work. Most regional EMS agencies have at least one paramedic staff on modified duty during a day shift. Modified paramedics remain in the employment of the paramedic service for the duration of their condition, but are often not using their skills and training in the community. Utilizing these paramedics for work they are physically able to do and fits within their skill set, is much more cost-effective and feasible than the placement of a family physician or other health practitioners, who would need to be funded from other external and potentially unavailable sources.

The main limitation of this study was that the intervention was only provided in one subsidized seniors’ building in Hamilton, Ontario. We do not know if the intervention would have been as successful in other buildings or in other areas with different residents and therefore different participants, with different resident death/move out rates (though we know these to be minimal from our communications with City Housing). A full evaluation, using a clustered randomized controlled trial (RCT) in multiple subsidized seniors’ buildings and in multiple urban areas around Ontario is planned to address this limitation [31].

CHAP-EMS is well aligned with evolving health policy and health care planning. Recent health policy is focused on the need to deliver innovative, community-based care with the dual goals of enabling older adults to live safely in their own homes and alleviating related pressures on more costly care settings such as acute care hospitals and long term care services [32]. For example, the Ministry of Health and Long Term Care in Ontario has recommended the exploration of the expansion of community paramedicine programs to support primary care access for older adults [30]. This is consistent with recommendations in other countries to utilize more community paramedicine [30, 33, 34]. The CHAP-EMS program is one community paramedicine approach that can be combined with others to help centre health care delivery in the community, primary care and home settings [35].

## Conclusions

The CHAP-EMS program, using an expanded role for community paramedicine, was a feasible approach to providing risk assessment and recommendations to address modifiable risk factors in high needs seniors living in an urban setting. Paramedics, more traditionally geared to emergency situations, are a relatively untapped source of community health support. The clinically meaningful decline in EMS calls and BP, and the tendency towards a decrease in CANRISK scores, are all encouraging and its benefits will be further evaluated in a larger RCT.

## Abbreviations

BP: Blood pressure
CANRISK: Canadian diabetes risk score
CBG: Capillary blood glucose
CCAC: Community care access centre
CHAP-EMS: Community health assessment program through emergency medical services
CVD: Cardiovascular disease
ED: Emergency department
EMS: Emergency medical services
RCT: Randomized control trial
TUG: Timed up and go test

## References

[1] Canadian Institute for Health Information. Seniors and the Health Care System: What Is the Impact of Multiple Chronic Conditions? [Internet]. 2011. Available from: https://secure.cihi.ca/free_products/air-chronic_disease_aib_en.pdf.

[2] Canadian Institute for Health Information. Pan-Canadian Forum for High Users of Health Care [Internet]. 2014. Available from: https://secure.cihi.ca/free_products/highusers_summary_report_revised_EN_web.pdf

[3] Platts-Mills TF, Leacock B, Cabañas JG, Shofer FS, McLean SA (2010). Emergency medical services use by the elderly: analysis of a statewide database. Prehosp Emerg Care.

[4] Svenson JE (2000). Patterns of use of emergency medical transport: a population-based study. Am J Emerg Med.

[5] Weiss SJ, Ernst AA, Miller P, Russell S (2002). Repeat EMS transports among elderly emergency department patients. Prehospital Emerg Care.

[6] Wofford JL, Moran WP, Heuser MD, Schwartz E, Velez R, Mittelmark MB (1995). Emergency medical transport of the elderly: a population-based study. Am J Emerg Med.

[7] Gibler KM (2003). Aging subsidized housing residents : a growing problem in U.S. Cities. J Real Estate Res.

[8] Rivera LA, Lebenbaum M, Rosella LC (2015). The influence of socioeconomic status on future risk for developing Type 2 diabetes in the Canadian population between 2011 and 2022: differential associations by sex. Int J Equity Health.

[9] Clark AM, DesMeules M, Luo W, Duncan AS, Wielgosz A (2009). Socioeconomic status and cardiovascular disease: risks and implications for care. Nat Rev Cardiol.

[10] Agarwal G, Angeles RN, McDonough B, McLeod B, Marzanek F, Pirrie M (2015). Development of a community health and wellness pilot in a subsidised seniors’ apartment building in Hamilton, Ontario: community health awareness program delivered by emergency medical services (CHAP-EMS). BMC Res Notes.

[11] Statistics Canada. Focus on Geography Series, 2011 Census [Internet]. 2012. Available from: https://www12.statcan.gc.ca/census-recensement/2011/as-sa/fogs-spg/Facts-csd-eng.cfm?LANG=Eng&GK=CSD&GC=3525005

[12] Hypertension Canada. What is High Blood Pressure? [Internet]. [cited 2016 Mar 26]. Available from: https://www.hypertension.ca/en/hypertension/what-do-i-need-to-know/what-is-high-blood-pressure

[13] Robinson CA, Agarwal G, Nerenberg K (2011). Validating the CANRISK prognostic model for assessing diabetes risk in Canada’s multi-ethnic population. Chronic Dis Inj Can.

[14] Kaczorowski J, Chambers Larry W, Dolovich L, Michael PJ, Karwalajtys T, Gierman T (2011). Improving cardiovascular health at population level: 39 community cluster randomised trial of Cardiovascular Health Awareness Program (CHAP). BMJ.

[15] Canadian Community Health Curvey: Statistics Canada. Accessed 5 Nov 2016: http://www23.statcan.gc.ca/imdb/p2SV.pl?Function=getSurvey&SDDS=3226

[16] Beauchet O, Fantino B, Allali G, Muir SW, Montero-Odasso M, Annweiler C (2011). Timed Up and Go test and risk of falls in older adults: a systematic review. J Nutr Health Aging.

[17] Havas S, Roccella EJ, Lenfant C (2004). Reducing the public health burden from elevated blood pressure levels in the United States by lowering intake of dietary sodium. Am J Public Health.

[18] Daskalopoulou SS, Rabi DM, Zarnke KB, Dasgupta K, Nerenberg K, Cloutier L (2016). The 2015 Canadian hypertension education program recommendations for blood pressure measurement, diagnosis, assessment of risk, prevention, and treatment of hypertension. Can J Cardiol.

[19] Emergency Health Services Branch OM of H and L-TC. Basic Life Support Patient Care Standards [Internet]. Toronto, ON; 2007. Available from: http://www.health.gov.on.ca/english/public%5Cprogram/ehs/edu/pdf/bls_patient.pdf

[20] Ontario Ministry of Health and Long-Term Care. Ambulance Services Billing [Internet]. [cited 2016 Mar 22]. Available from: http://www.health.gov.on.ca/en/public/publications/ohip/amb.aspx

[21] Hamilton Spectator. Code red: Band-Aid fixes getting us nowhere. Hamilt. Spect. [Internet]. Hamilton; [cited 2015 Feb 10]; Available from: http://www.thespec.com/news/article/21318--code-red-band-aid-fixes-getting-us-nowhere

[22] Canadian Institute for Health Information. Sources of Potentially Avoidable Emergency Department Visits [Internet]. 2014. Available from: https://secure.cihi.ca/free_products/ED_Report_ForWeb_EN_Final.pdf

[23] Home Care Ontario. Home Care Services: Facts and Figures- Publicly Funded Home Care [Internet]. Available from: http://www.homecareontario.ca/home-care-services/facts-figures/publiclyfundedhomecare

[24] World Health Organization. Noncommunicable diseases country profiles 2011 [Internet]. 2011. Available from: http://www.who.int/nmh/publications/ncd_profiles2011/en/

[25] Public Health Agency of Canada. Report from the Canadian chronic disease surveillance system: Hypertension in Canada [Internet]. Ottawa; 2010. Available from: http://www.phac-aspc.gc.ca/cd-mc/cvd-mcv/ccdss-snsmc-2010/pdf/CCDSS_HTN_Report_FINAL_EN_20100513.pdf

[26] Public Health Agency of Canada. Diabetes in Canada: Facts and figures from a public health perspective [Internet]. Ottawa, ON; 2011. Available from: http://www.phac-aspc.gc.ca/cd-mc/publications/diabetes-diabete/facts-figures-faits-chiffres-2011/highlights-saillants-eng.php

[27] Canadian Diabetes Association. 2008 Clinical Practice Guidelines | Executive Summary | February 2009 1 Canadian Diabetes Association 2008 Clinical Practice Guidelines for the Prevention and Management of Diabetes in Canada: Executive Summary [Internet]. 2009. Available from: http://archive.diabetes.ca/files/for-professionals/CPGExecSummaryEssentials.pdf

[28] Tran N, McGuire H. Smoke-Free Ontario Strategy Evaluation [Internet]. Hamilton, ON; 2012. Available from: http://www2.hamilton.ca/NR/rdonlyres/50BED0D2-15CA-409D-93F5-9BB639DE1AF3/0/Jun1851EDRMS_n319644_v1_BOH12012__SmokeFree_Ontario_Strategy_Ev.pdf

[29] Merzel C, D’afflitti J (2003). Reconsidering community-based health promotion: promise, performance, and potential. Am J Public Health.

[30] Bigham BL, Kennedy SM, Drennan I, Morrison LJ (2013). Expanding paramedic scope of practice in the community: a systematic review of the literature. Prehospital Emerg Care.

[31] Agarwal G, McDonough B, Angeles R, Pirrie M, Marzanek F, McLeod B (2015). Rationale and methods of a multicentre randomised controlled trial of the effectiveness of a Community Health Assessment Programme with Emergency Medical Services (CHAP-EMS) implemented on residents aged 55 years and older in subsidised seniors’ housing b. BMJ Open.

[32] Sinha S. Living longer, Living well: Recommendations to Inform a Seniors Strategy for Ontario [Internet]. 2012. Available from: http://healthcareathome.ca/centraleast/en/news/Documents/Seniors_Strategy.pdf.

[33] O’Meara P (2003). Would a prehospital practitioner model improve patient care in rural Australia?. Emerg Med J.

[34] Mason S, Wardrope J, Perrin J (2003). Developing a community paramedic practitioner intermediate care support scheme for older people with minor conditions. Emerg Med J.

[35] Iezzoni LI, Dorner SC, Ajayi T (2016). Community paramedicine—addressing questions as programs expand. N Engl J Med Mass Medical Soc.

